# Circular RNA Related to the Chondrocyte ECM Regulates MMP13 Expression by Functioning as a MiR-136 ‘Sponge’ in Human Cartilage Degradation

**DOI:** 10.1038/srep22572

**Published:** 2016-03-02

**Authors:** Qiang Liu, Xin Zhang, Xiaoqing Hu, Linghui Dai, Xin Fu, Jiying Zhang, Yingfang Ao

**Affiliations:** 1 Institute of Sports Medicine, Beijing Key Laboratory of Sports Injuries, Peking University Third Hospital, 49 North Garden Road, Haidian District, Beijing 100191, P. R. China

## Abstract

Circular RNAs (circRNAs) are involved in the development of various diseases, but there is little knowledge of circRNAs in osteoarthritis (OA). The aim of study was to identify circRNA expression in articular cartilage and to explore the function of chondrocyte extracellular matrix (ECM)-related circRNAs (circRNA-CER) in cartilage. To identify circRNAs that are specifically expressed in cartilage, we compared the expression of circRNAs in OA cartilage with that in normal cartilage. Bioinformatics was employed to predict the interaction of circRNAs and mRNAs in cartilage. Loss-of-function and rescue experiments for circRNA-CER were performed *in vitro*. A total of 71 circRNAs were differentially expressed in OA and normal cartilage. CircRNA-CER expression increased with interleukin-1 and tumor necrosis factor levels in chondrocytes. Silencing of circRNA-CER using small interfering RNA suppressed MMP13 expression and increased ECM formation. CircRNA-CER could compete for miR-136 with MMP13. Our results demonstrated that circRNA-CER regulated MMP13 expression by functioning as a competing endogenous RNA (ceRNA) and participated in the process of chondrocyte ECM degradation. We propose that circRNA-CER could be used as a potential target in OA therapy.

Osteoarthritis (OA) is a degenerative joint disease characterized by articular cartilage degradation, subchondral bone thickening, and osteophyte formation^1^,^2^. Cartilage cellularity is reduced in OA by chondrocyte death; chondrocytes are stimulated by cytokines and growth factors to undergo catabolic and abnormal differentiation, leading to extracellular matrix (ECM) degradation^3^,^4^,^5^,^6^. The molecular mechanisms involved in the maintenance of articular cartilage have been characterized to develop new therapeutic interventions^7^.

Circular RNAs (circRNAs) are a large class of non-coding RNAs that exist ubiquitously in the cytoplasm of eukaryotic cells^8^,^9^; these endogenous RNAs are characterized by a stable structure and high tissue-specific expression^10^. Compared with linear RNAs, circRNAs exhibit the remarkable characteristic of undergoing non-canonical splicing without a free 3′ or 5′ end^11^,^12^. Recently, it was reported that circRNAs function as miRNA ‘sponges’ that naturally sequester and competitively suppress miRNA activity^13^. It was demonstrated that circRNAs are involved in the development of several types of diseases, such as atherosclerosis and nervous system disorders^14^,^15^,^16^. However, the role of circRNAs in cartilage and their overall contribution to OA pathogenesis are still unknown.

In the present study, we identified a small number of circRNAs that are up- or down-regulated in OA versus normal cartilage. These circRNAs may exhibit an important function in cartilage injury and OA development and progression. We examined the functions and mechanisms of circRNAs in OA pathogenesis. We initially identified a new circRNA involved in the process of cartilage injury and proposed that circRNA-CER could be used as a potential target in OA therapy.

## Results

### Profile of circRNA expression in cartilage

**Figure 1:**
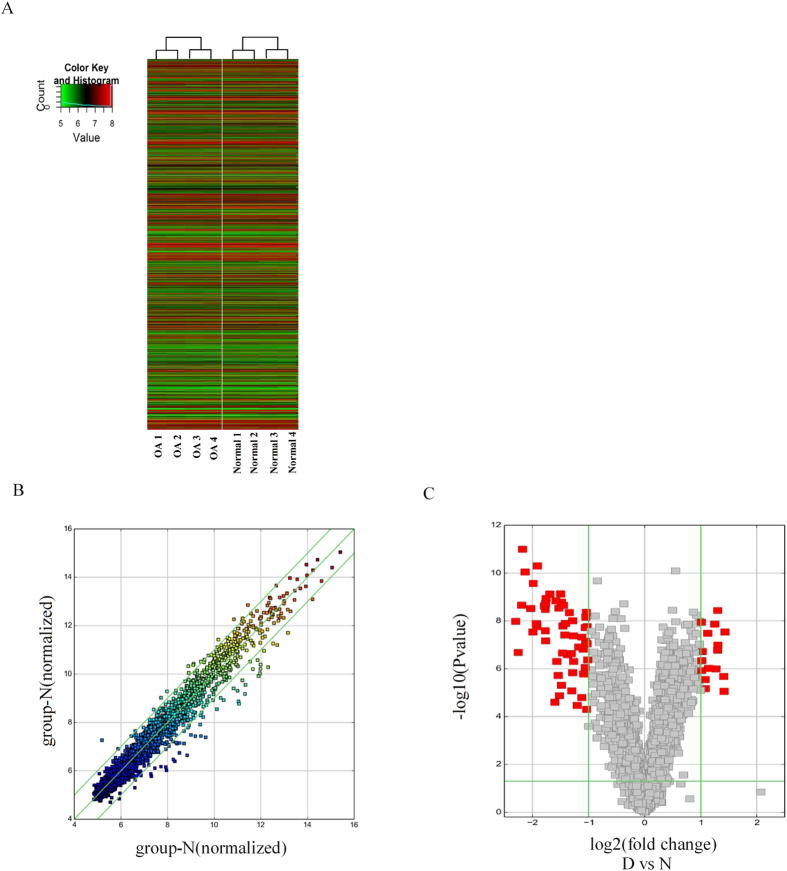
Differential expression of circRNAs in cartilage tissues. (**A**) Hierarchical clustering analysis of circRNAs that were differentially expressed between OA and normal cartilage samples; each group contains 4 individuals (greater than twofold difference in expression; P < 0.05). Expression values are represented in different colors, indicating expression levels above and below the median expression level across all samples. (**B**) The scatter plot is a visualization method used for assessing the variation in circRNA expression between normal (group-N) and OA (group-D) samples. The values corresponding to the X- and Y-axes in the scatter plot are the normalized signal values of the samples (log2 scaled). The green lines indicate fold changes. The circRNAs above the top green line and below the bottom green line indicate more than 2.0-fold changes between the normal and OA samples. (**C**) Volcano plots were constructed using fold-change values and p-values. The vertical lines correspond to 2.0-fold up- and down-regulation between normal and OA samples (D vs N), and the horizontal line represents a *P*-value. The red point in the plot represents the differentially expressed circRNAs with statistical significance.

**Figure 2:**
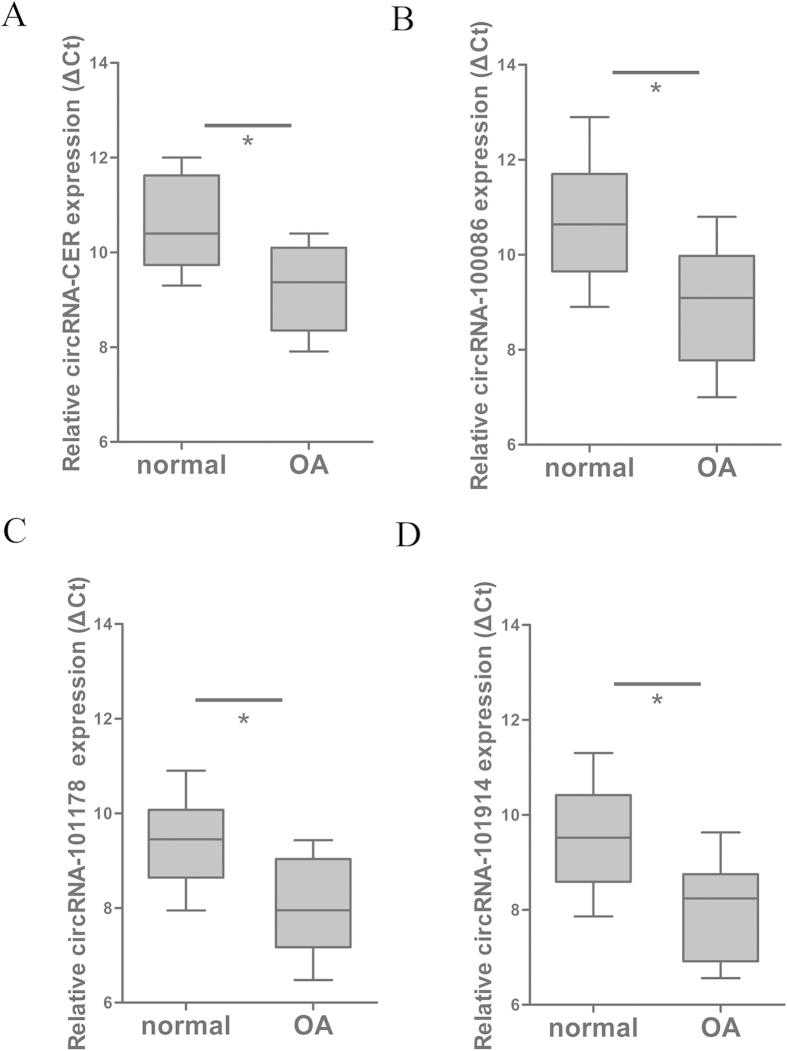
Validation of the differential expression of four circRNAs. The expression levels of the following circRNAs were analyzed via qPCR: circRNA_100086, circRNA_101178, circRNA_101914 and circRNA_100876 (circRNA-CER). ΔCt values were used to measure gene expression, which was normalized according to GAPDH expression levels. Normal group = 10, OA group = 20. The presented values are the means ± SEM. *P < 0.05.

Hierarchical clustering revealed circRNA expression in normal and OA cartilage samples (Fig. 1A). The scatter and volcano plots showed the variation of circRNA expression between the normal and OA cartilage samples (Fig. 1B,C). We identified 71 circRNAs that were differentially expressed in OA versus normal cartilage. Sixteen of these circRNAs were up-regulated and 55 were down-regulated in OA tissues. To validate the circRNA microarray results, we selected four circRNAs that exhibited significant changes in expression among the differentially expressed circRNAs and then performed qPCR to analyze the changes in expression. The data confirmed that circRNA_100876 (circRNA-CER), circRNA_100086, circRNA_101178 and circRNA_101914 were overexpressed in OA. The circRNA-CER was up-regulated in OA with 2.5-fold change by qPCR (Fig. 2).

### Prediction of circRNAs related to cartilage and target genes

**Figure 3:**
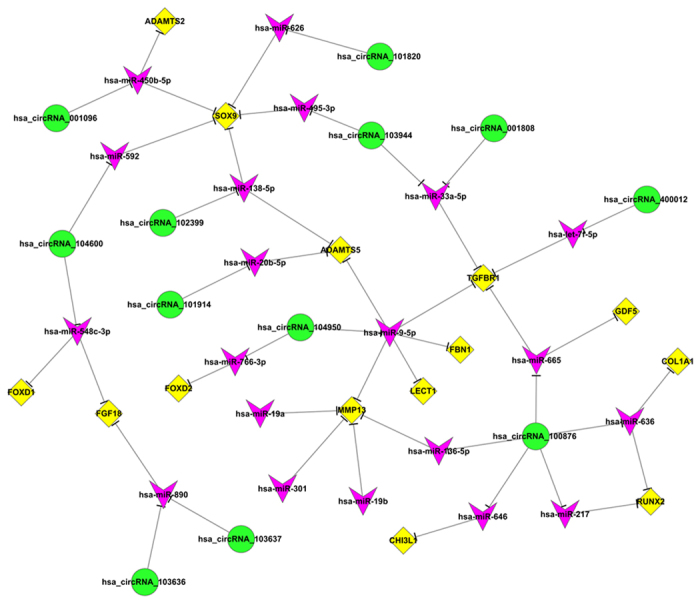
circRNAs-miRNAs-mRNAs network. The network consists of 46 genes. In the CircRNA-miRNA-mRNA-Net, the circle represents circRNA and the shape of rhombus represents mRNA and the shape of inverted triangle represents miRNA, and their relationship was represented by one edge.

**Figure 4:**
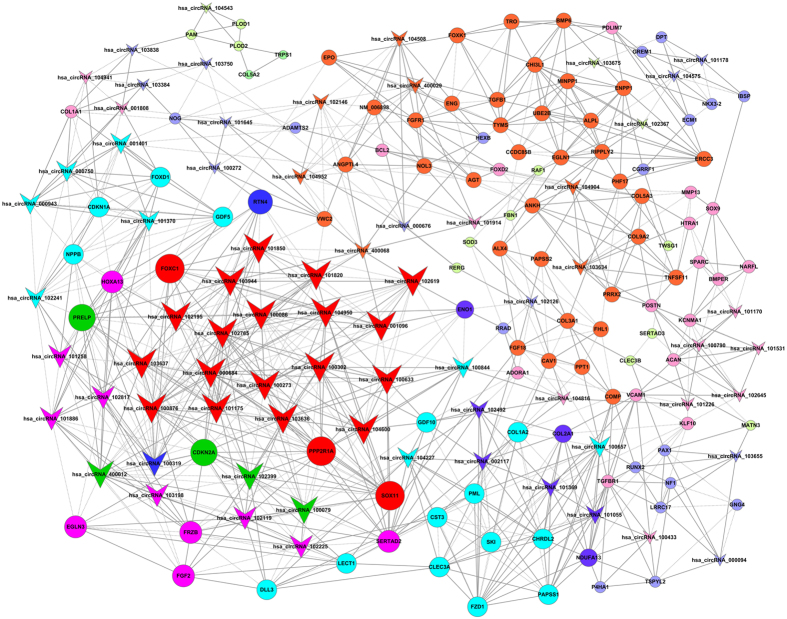
Co-expression network in OA cartilage. The co-expression network consists of 183 genes. A round node represents a protein-coding gene, and a triangular node represents a circRNA. Solid lines between two nodes indicate positively correlated interactions between genes, and dashed lines indicate negatively correlated interactions.

**Figure 5:**
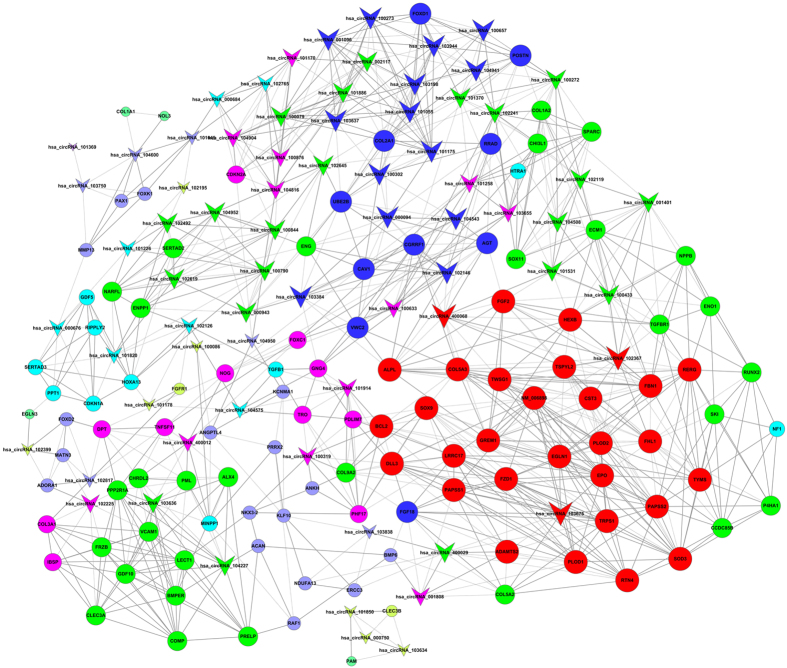
Co-expression network in normal cartilage. The co-expression network consists of 183 genes. A round node represents a circRNA, and a triangular node represents a protein-coding gene. Solid lines between two nodes indicate positively correlated interactions between genes, and dashed lines indicate negatively correlated interactions.

We used gene co-expression networks to predict the circRNA targets. The structures of the co-expression networks were constructed according to the normalized signal intensities of circRNAs and mRNAs in OA and normal cartilage. There are a total of 71 circRNAs and 112 mRNAs in the network. In these co-expression networks, each gene corresponds to a node, and 2 genes are connected by a string, indicating a tight correlation between those genes and a potential regulatory relationship (Figs 3 and 4). Furthermore, we constructed another network according to the common miRNAs binding circRNAs and mRNAs. The differentially expressed circRNAs were annotated in detail with the miRNA interaction information (Supplementary Table 1). We selected cartilage-related mRNAs and predicted their binding miRNAs. Through merging the common targeted miRNAs, we constructed a network of circRNAs-miRNAs-mRNAs with a total of 12 circRNAs, 14 mRNAs and 20 miRNAs. (Fig. 5). Each gene corresponded to a node, and 2 genes were connected by a string. It indicated the tight correlation and regulation relationship of these genes. In the network, circRNA-CER was connected by 5 miRNAs.

### Up-regulation of novel circRNA-CER in chondrocytes stimulated with IL-1 and TNFα

**Figure 6:**
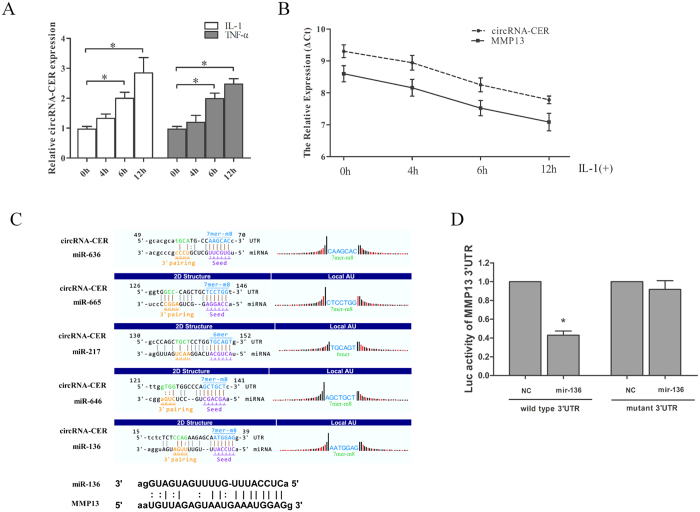
Gene expression changes following IL-1 and TNF-α treatment and circRNA-CER-targeting microRNAs. (**A**) Chondrocytes from normal donors were treated with IL-1 and TNF-α for the indicated times, and circRNA-CER expression was analyzed via qPCR after IL-1 and TNF-α treatment for 4, 6, and 12 h compared with 0 h. (**B**) circRNA-CER and MMP13 expression levels in chondrocytes increased with IL-1 treatment duration. ΔCt values were used to measure gene expression, which was normalized according to GAPDH expression levels. The presented values are the mean ± SEM of 3 different preparations, *P < 0.05. (**C**) Targeted microRNAs matched circRNA-CER and MMP13 3′UTRs. MicroRNAs binding to MMP13 and circRNA-CER matched by solid lines. (**D**) Luciferase reporter analysis of either wild-type or mutant MMP13 3′-UTR activity. MiR-136 was co-transfected with the wild-type or mutant vector. The presented values are the mean ± SEM of 3 different preparations *P < 0.05.

We assumed that circRNA-CER was a special chondrocyte ECM-related circRNA(ID: hsa_circ_0023404 in CircBase: http://circbase.org/cgi-bin/simplesearch.cgi). Its gene is located at chr11:71668272-71671937, and its associated-gene symbol is RNF121. The length of the circRNA-CER is 180 bp. The circRNA-CER was chosen for it is one of the circRNAs indicated to be associated with cartilage according to bioinformatics analysis mentioned above. Catabolic stimulators, such as IL-1 and TNFα, are critical mediators of OA, and stimulation of chondrocytes with these inflammatory mediators resulted in a similar pattern of gene expression to that observed in OA^17^. This phenomenon results in down-regulation of ECM genes and up-regulation of MMP13 and ADAMTS5^18^. circRNA-CER expression was also increased with the duration of IL-1 and TNFα treatment (Fig. 6A). Moreover, circRNA-CER expression paralleled MMP13 expression under stimulation with the inflammatory mediators (Fig. 6B). Overall, the results demonstrated co-regulation of circRNA-CIR and MMP13 under catabolic *in vitro* conditions.

### CircRNA-CER is targeted by MMP13-targeting miRNAs

It has been reported that circRNAs function as miRNA ‘sponges’ that naturally sequester and competitively suppress miRNA activity^13^. We assumed that circRNA-CER functions as a decoy to regulate MMP13 expression through the same mechanism. According to the network above, there were 5 miRNA binding sites for circRNA-CER, and they were miR-636, miR-665, miR-217, miR-646 and miR-136, respectively. The sequence of the circRNA-CER 3′UTR matched these miRNAs. We identified common miRNAs for the circRNA-CER and MMP13 targets through informatics analysis. The result showed that miR-136 also matched the 3′UTR of MMP13 (Fig. 6C). The miR-Report luciferase reporter was constructed to determine whether miRNA can directly target the 3′-UTR of MMP13. The reporter was co-transfected with miR-135 mimics. The luciferase signal of the wild-type MMP13 reporter was suppressed by miR-136, whereas the luciferase signal of the mutant reporter was not affected (Fig. 6D).

### Effects of circRNA-CER on MMP 13 expression as a ceRNA in chondrocyte degradation

To analyze the effects of circRNA-CER on ECM degradation, we examined the effect of knockdown of circRNA-CER in OA chondrocytes. The siRNA used in this experiment was specific for circRNA-CER. As a consequence of this inhibition, the expression of mRNA for MMP13 decreased, whereas the expression of mRNA for COL2 and aggrecan increased.

**Figure 7:**
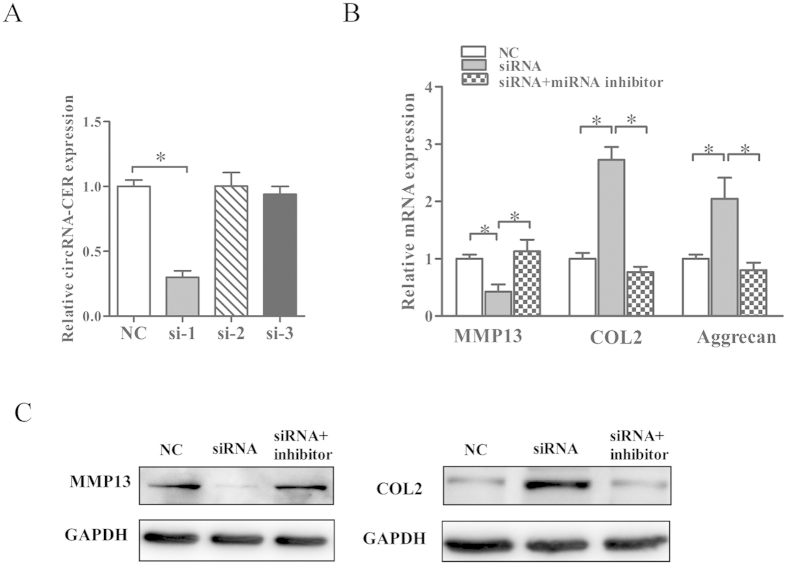
Effects of circRNA-CER on gene expression in human chondrocytes. (**A**) The effect of 3 different small interfering RNAs (siRNAs) against circRNA-CER (si-CER) was analyzed by quantitative polymerase chain reaction (qPCR). The best inhibitory result was achieved by si-CER-1 (si-1). (**B**) MMP13, COL2 and aggrecan mRNA expression levels were detected following knockdown of circRNA-CER using si-CER or co-transfection with si-CER and the miR-136 inhibitor. (**C**) MMP13 and COL2 protein expression levels were analyzed by western blotting. GAPDH was used as a loading control. The presented values are the mean ± SEM of 3 different preparations *P < 0.05.

We inferred that circRNA-CER induced ECM degradation and regulated MMP13 expression by functioning as a ceRNA. We co-transfected the miR-136 inhibitor and si-CER into chondrocytes. We confirmed that MMP13 repression via circRNA-CER knockdown was reversed by the miR-136 inhibitor, and the effect regarding the high expression of COL2 and aggrecan was also eliminated (Fig. 7A).

**Figure 8:**
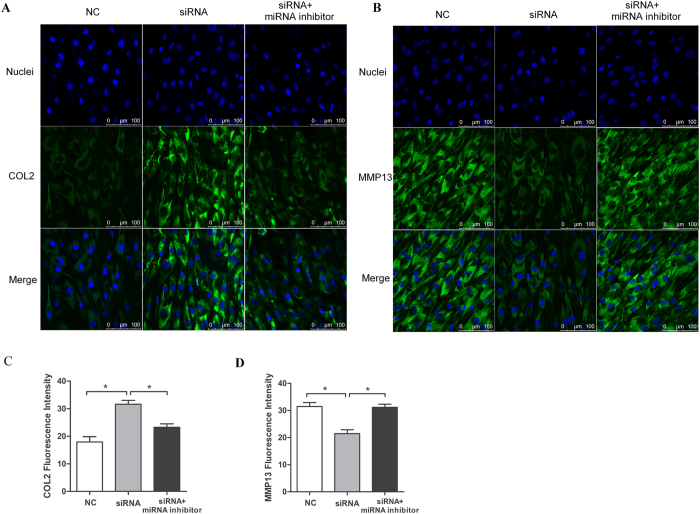
Immunofluorescence analysis of COL2 and MMP13 expression in chondrocytes. (**A,B**) Immunofluorescence staining for COL2 and MMP13 after transfection with si-CER or co-transfection with si-CER and the miR-136 inhibitor. Specific antibodies against COL2 and MMP13 were used, together with fluorescein isothiocyanate and Hoechst 33342 staining. Scale bars = 100 μm. (**C,D**) The fluorescence intensities of the respective images in A and B were quantified using Image-Pro Plus 6.0 software. The presented values are the mean ± SEM of at least 3 independent experiments. *P < 0.05.

We performed western blotting to examine the expression of COL2 and MMP13 at the protein level. Type II collagen protein expression was significantly elevated by si-CER treatment, and this increase was reversed through co-transfection with the miR-136 inhibitor and si-CER in chondrocytes. Additionally, MMP-13 protein expression was significantly increased by si-CER treatment, and this effect was reduced by co-transfection with the miR-136 inhibitor and si-CER in chondrocytes (Fig. 7B). ECM proteins were also evaluated through immunofluorescence staining, and the results were consistent with the qPCR and western blot analyses (Fig. 8).

## Discussion

Many studies on OA have focused on the epigenetic regulation of its pathogenesis and potential targets for therapy, including microRNAs and long noncoding RNAs (lncRNAs). However, the occurrence of circRNAs in cartilage remains largely unknown. This study is the first to profile circRNA expression in human cartilage tissue. In this study, we identified a number of circRNAs that are aberrantly expressed in OA compared with normal cartilage. Through loss-of-function and rescue experiments, we found that circRNA-CER is important in the process of ECM degradation in chondrocytes.

Recent studies have shown that many exonic transcripts can form circRNAs through non-linear reverse splicing or gene rearrangement^8^. CircRNAs may arise from exons or introns. Both exonic and intronic circRNAs show potential functions in the regulation of gene expression^19^. CircRNAs are widely expressed in human cells, and their expression levels can be higher by 10-fold or more compared with their linear isomers^20^. Two properties of circRNAs are the most important: first, they are highly conserved sequences; second, they show a high degree of stability in mammalian cells^13^. Compared with other noncoding RNAs, such as miRNAs and lncRNAs, these properties provide circRNAs with the potential to be used as ideal biomarkers and potential therapy targets^21^. It has been reported that the antisense sequence for the cerebellar degeneration-related protein1 transcript (CDR1as) is a representative circRNA containing approximately 70 binding sites for miR-7, and it is not easily degraded by the RNA-induced silencing complex (RISC). Therefore, this sequence plays a role as a miRNA sponge^11^,^22^.

We assumed that circRNA-CER functioned as a decoy to regulate MMP13 expression through the same mechanism. We found that circRNA-CER harbors miRNA-binding sites, including miR-636, miR-665, miR-217, miR-646 and miR-136. In addition, miR-136 could also bind to the 3′-UTR of MMP13. Several studies reported that miR-136 might play important roles in regulating chondrogenic differentiation of human Adipose-Derived Stem Cell and affect the process of chondrogenesis^23^. It was also reported that miR-217 inhibit Runx2 protein expression in osteoblasts and chondrocytes, it also significantly impeded osteoblast differentiation, and the effect could be reversed by the corresponding anti-miRNA^24^,^25^. These findings indicated that the mechanism of circRNAs was complicated and many other molecules participated in the process of cartilage degradation. In our study, we only focused on miR-136, as the only miRNA could target both circRNA-CER and MMP13. However, due to the limited number of samples of normal cartilage tissues available, we only analyzed circRNA-CER expression in 30 tissues from donors, and additional samples should be analyzed in the future. Also, the functions of circRNA-CER *in vivo* need to be confirmed by our further studies.

In our study, we constructed a network including circRNAs and mRNAs and a circRNA-miRNA-mRNA network. These two networks indicated the potential associations between circRNAs and their target genes. Meanwhile, the networks provided an important reference value for studying the interaction of other differential expressed circRNAs and their potential targets.

In our study, we demonstrated that circRNA-CER regulated MMP13 expression and participated in the process of chondrocyte ECM degradation. There are multiple signaling pathways involved in this process. One of these superfamily is TGFβ. It is crucial for joint development and homeostasis and have been implicated in the pathogenesis of OA^26^. In micromass culture, TGFβ treatment delayed chondrocyte maturation and hypertrophy, and inhibited expression of type X collagen, VEGF and MMP13^27^. It has also been shown that TGFβ can activate canonical BMP pathways through engagement of ALK1 and that this pathway cause activation of Smads1/5/8 in cartilage^28^,^29^. And there is a significant correlation between ALK1 and MMP13 expression in OA cartilage^30^.

High level of activated JNK is seen in OA cartilage. It showed that inhibiting JNK could block MMP13 expression in human chondrocytes^31^,^32^. Chondrocytes also express both ERK1 and ERK2. ERK also plays a role in stimulating MMP13 expression in human chondrocytes, and inhibiting ERK prevents MMP13 expression. In summary, TGFβ, JNK and ERK pathways have different functions but they all could target MMP13 in regulating chondrocyte, so circRNA-CER may affect these pathways by regulation MMP13.

We confirmed that silencing of circRNA-CER by siRNA could suppress MMP13 expression and increased ECM formation. So circRNA-CER could be used as a potential target and specific siRNA used as therapeutic agents in OA therapy. The most attractive aspect of this therapeutics is their ability to target gene(s), which may not be possible with small molecules or protein-based drugs^33^. And it opens up a whole new therapeutic approach for the treatment of osteoarthritis by targeting genes that are involved causally in the pathological process.

Collectively, our data indicate that 71 circRNAs were either over- or under-expressed in OA. It has been suggested that the observed changes have biologic effects and that circRNAs are key regulators of gene expression. We confirmed that circRNA-CER is the decoy for MMP13 and that circRNA-CER functions like a “sponge” by competitively binding miR-136. The mechanism needs to be confirmed with further specific studies. Deciphering the precise molecular mechanisms of circRNA function in OA will be critical for understanding OA pathogenesis and exploring new potential therapeutic targets.

## Materials and Methods

### Patients and specimens

OA cartilage was isolated from the knee joints of 20 patients undergoing total knee arthroplasty (7 men and 13 women; age range 57–73 years), and normal articular cartilage was isolated from the knee joints of 10 donors after death or from trauma patients (5 men and 5 women; age range 29–65 years). All tissues were processed to be examined histologically and were graded according to the modified Mankin scale^34^. All of the tissue donors included in this study provided their informed consent. The study was approved by the Human Ethics Committee of the Peking University Third Hospital (China). Methods were carried out in “accordance” with the approved guidelines.

### Histological examination

The cartilage was removed and fixed in 4% paraformaldehyde in PBS solution and then demineralized in 15% EDTA. The cartilage specimens were dehydrated in a graded series of alcohol and xylene, then embedded in paraffin and cut serially into 5-mm sagittal sections. The sections were stained with Toluidine blue, Safranin-O, and hematoxylin and eosin (H&E) as per routine protocol. Changes were graded according to a modified Mankin scale^34^. A score of <2 points was considered normal, and a score of >5 represented OA^35^.

### Microarray and quantitative analysis

Joint tissue was immediately shock-frozen in liquid nitrogen, and within 24 hours, the articular cartilage was isolated from the condyles and tibia plateaus using a surgical blade^18^. Next, the samples (4 OA and 4 normal) were homogenized in TRIzol reagent (Invitrogen), and the total RNA in each sample was quantified using a NanoDrop ND-1000. Sample preparation and microarray hybridization were performed based on the Arraystar standard protocols. Briefly, total RNA from each sample was amplified and transcribed into fluorescent cRNA utilizing random primers according to the Arraystar Super RNA Labeling protocol (Arraystar Inc.). The labeled cRNAs were hybridized onto the Arraystar Human circRNA Array (8 × 15 K, Arraystar). After washing the slides, the arrays were scanned with an Agilent G2505C Scanner. Agilent Feature Extraction software (version 11.0.1.1) was used to analyze the acquired array images. Quantile normalization and subsequent data processing were performed with the R software package.

### Bioinformatics analysis

Differentially expressed mRNAs were associated with gene ontology analysis According to the enrichment analyses of gene ontology, we selected specific mRNAs to construct the network according to our previous microarray data^18^. The circRNAs were selected according to the circRNA profiling data. Co-expression networks were constructed according to the normalized signal intensities of circRNAs and mRNAs in the original microarray data^36^. Pearson’s correlation analysis was applied to measure the significance of the correlation of expression between each gene pair. When the expression levels of 2 genes were similar above a preselected threshold in the Pearson analysis, they were considered to exhibit a co-expression relationship and would be connected. Each gene corresponded to a node, and 2 genes were connected by a string, indicating a tight correlation. The degree of correlation was determined genes importance in the network^37^,^38^. An mRNA-miRNA-circRNA network was constructed according to the common target miRNAs of the circRNAs and mRNAs. The interactions of circRNAs and mRNAs with miRNAs were predicted with the Arraystar miRNA target prediction software based on TargetScan and miRanda^39^,^40^.

### Interleukin-1 (IL-1) and tumor necrosis factor-α (TNF-α) stimulation of human chondrocytes

Chondrocytes from OA patients and normal donors were isolated as described above^18^ and digested first with 0.25% trypsin (Invitrogen) for 30 minutes and then with 0.2% type II collagenase (Invitrogen) for 4–6 hours at 37 °C. The chondrocytes were maintained in Dulbecco’s modified Eagle’s medium (DMEM; Invitrogen) supplemented with 10% fetal bovine serum (HyClone), 100 units/ml of penicillin, and 100 units/ml of streptomycin. The chondrocytes were stimulated by the addition of 10 ng/ml of IL-1 or TNF-α (PeproTech) in the culture medium for 4, 6, or 12 hours. Unstimulated chondrocytes were used as controls. The controls were treated in exactly the same manner as the stimulated chondrocytes, except that IL-1/ TNF-α was not added.

### Luciferase assay

MMP13 3′-UTR containing the putative binding site of miR-136, as well as its identical sequence with a mutation of the miR-136 seed sequence, was inserted between the restrictive sites Xho I and Not I of pmiR-RB-ReportTM and validated by sequencing. HeLa cells were transfected with wild-type or mutated reporter vectors, miRNA mimics, and negative control. Lysates were harvested 24 h after transfection. Renilla luciferase activities were consecutively measured according to the dual-luciferase assay manual (Promega).

### RNA interference and transfection

Small interfering RNAs (siRNAs) targeting circRNA-CER (referred to as si-circCERs) were designed and synthesized by RiboBio. The sequence of the functional si-lncCIR is CCCACGCTCCTACAATGTT. Chondrocytes were transfected with si-circCERs using Lipofectamine 2000 (Invitrogen) according to the manufacturer’s protocol. Briefly, the cells were cultured with DMEM in a 96-well plate as described above. Prior to transfection, the culture medium was replaced with medium without antibiotics, and the cells were cultured for 24 hours. The RNA interference Lipofectamine 2000 complex was prepared by mixing for 20 minutes, and the complex was then added to each well. The cells were cultured for 48 hours at 37 °C with normal DMEM.

### Real-time PCR

Total RNA was isolated from cartilage tissues or monolayer-cultured primary chondrocytes using TRIzol reagent. For miRNA quantitative PCR analysis, reverse transcription of specific miRNAs was performed with the Bulge-Loop™ miRNA Primer Set (RiboBio) according to the manufacturer’s instructions. For mRNA analysis, total RNA was reverse transcribed using random primers. mRNA expression levels are reported relative to glyceraldehyde 3-phosphate dehydrogenase (GAPDH), while miRNA expression levels are reported relative to U6. The primers used in the present study were as follows:

COL2 forward: 5′-TGGACGATCACGAAACC-3′, reverse: 5′-GCTGCGGATGCTCTCAATCT-3′;

aggrecan forward: 5′-ACTCTGGGTTTTCGTGACTCT-3′, reverse: 5′-ACACTCAGCGAGTTGTCATGG-3′;

MMP13 forward: 5′-ACTGAGAGGCTCCGAGAAATG-3′, reverse: 5′-GAACCCCGCATCTTGGCTT-3′;

circRNA-CER forward: 5′-CTGGTGCAGTGGAAGCAGAG-3′, reverse: 5′- CGACCCTCCATTGCTCTTCT -3′;

hsa_circRNA_100086 forward: 5′-CCATCCCCTTATTCAGCACAT-3′, reverse: 5′-TCCAAACTTCAGTTTCCTCATCA-3′;

hsa_circRNA_101178 forward: 5′-CTGCGTTGGGAGTCTGGAAG-3′, reverse: 5′- TCACAGGTGAGTGGCAAGTGAG -3′;

GAPDH forward: 5′-GGGAAACTGTGGCGTGAT-3′, reverse: 5′- GAGTGGGTGTCGCTGTTGA -3′;

### Western blotting

Protein was extracted using lysis buffer (50 mM Tris HCl, pH 7.4, 150 mM NaCl, 1% Nonidet P40, and 0.1% sodium dodecyl sulfate). The concentration was determined using a bicinchoninic acid (BCA) protein assay kit (Pierce). Proteins were run on sodium dodecyl sulfate–polyacrylamide gel electrophoresis gels (10%) and were electro-transferred to a nitrocellulose membrane for 2 hours at 4 °C. The blots were probed overnight at 4 °C with antibodies against COL2 (1:1,000 dilution; Abcam) and MMP13 (1:3,000 dilution; Abcam), followed by incubation for 1 h at room temperature with a horseradish peroxidase-conjugated secondary antibody (1:4,000 dilution; Santa Cruz Biotechnology). Proteins were detected via chemiluminescence according to the manufacturer’s recommendations (ECL Millipore). GAPDH was used as an internal control.

### Immunofluorescence analysis

The cultured cells were rinsed in PBS and fixed with 4% paraformaldehyde for 15 minutes at room temperature. Goat serum was used to block nonspecific binding sites. The cultured cells were incubated with anti-COL2 (1:200 dilution) and anti-MMP13 (1:200 dilution) at 4 °C overnight. The cells were subsequently incubated for 1 hour with fluorescein isothiocyanate–conjugated AffiniPure goat anti-rabbit IgG (1:100 dilution). Finally, the samples were incubated for 5 minutes with Hoechst 33342 and observed with a confocal microscope (FV 1000 Olympus IX-81). Images were analyzed using Image-Pro Plus 6.0 software (Media Cybernetics).

### Statistical analysis

Statistically significant differences from multiple groups were calculated through analysis of variance (ANOVA) and S-N-K post-hoc test was performed after ANOVA. Results from two group were evaluated using t-tests. The results are reported as the mean ± SEM. *P* values of less than 0.05 were considered statistically significant. All experiments were performed and analyzed in triplicate. Data analysis was performed with SPSS software.

## Additional Information

**How to cite this article**: Liu, Q. *et al*. Circular RNA Related to the Chondrocyte ECM Regulates MMP13 Expression by Functioning as a MiR-136 ‘Sponge’ in Human Cartilage Degradation. *Sci. Rep*. **6**, 22572; doi: 10.1038/srep22572 (2016).

## Supplementary Material

Supplementary Information
