# Supplementary material for: Circular RNA Related to the Chondrocyte ECM Regulates MMP13 Expression by Functioning as a MiR-136 ‘Sponge’ in Human Cartilage Degradation
Source: Sci Rep. 2016 Mar 2;6:22572. doi: 10.1038/srep22572 (PMC4773870; doi:10.1038/srep22572)
Supplement: Supplementary Information [file srep22572-s1.pdf]

Circular RNA Related to the Chondrocyte ECM Regulates MMP13 Expression by Functioning as a MiR-136 'Sponge' in Human Cartilage Degradation

Qiang Liu[1], Xin Zhang[1], Xiaoqing Hu[1], Linghui Dai[1], Xin Fu[1], Jiying Zhang[1], Yingfang Ao[1*]

1 Institute of Sports Medicine, Beijing Key Laboratory of Sports Injuries, Peking University Third Hospital, 49 North Garden Road, Haidian District, Beijing 100191, P. R. China.

Correspondence authors' Address:

*Institute of Sports Medicine, Peking University Third Hospital, 49 North Garden Road, Haidian District, Beijing 100191, P. R. China. (Yingfang Ao, MD)

Fax number: 86-10-62010440; Tel. 86-10-82267390; Email: yingfang.ao@gmail.com;

circRNA	Fold change	MRE1	MRE2	MRE3	MRE4	MRE5
hsa_circRNA_000684	up 2.4672931	hsa-miR-30d-3p	hsa-miR-185-5p	hsa-miR-30e-3p	hsa-let-7b-5p	hsa-let-7c-5p
hsa_circRNA_001096	up 2.1133125	hsa-miR-450b-5p	hsa-miR-210-5p	hsa-miR-19b-1-5p	hsa-miR-198	hsa-miR-525-5p
hsa_circRNA_001808	up 2.0355784	hsa-miR-33b-5p	hsa-miR-93-3p	hsa-miR-33a-5p		
hsa_circRNA_100086	up 2.6979508	hsa-miR-23b-5p	hsa-miR-581	hsa-miR-23a-5p	hsa-miR-450a-1-3p	hsa-miR-146a-3p
hsa_circRNA_100876	up 2.023046	hsa-miR-636	hsa-miR-665	hsa-miR-217	hsa-miR-136-5p	hsa-miR-646
hsa_circRNA_101178	up 2.1802434	hsa-miR-422a	hsa-miR-874-3p	hsa-miR-765	hsa-miR-552-3p	hsa-miR-516a-5p
hsa_circRNA_101820	up 2.0144796	hsa-miR-19a-3p	hsa-miR-19b-3p	hsa-miR-301a-3p	hsa-miR-626	hsa-miR-519c-3p
hsa_circRNA_101914	up 2.465095	hsa-miR-766-5p	hsa-miR-298	hsa-miR-345-3p	hsa-miR-490-5p	hsa-miR-20b-5p
hsa_circRNA_102241	up 2.3768615	hsa-miR-323a-5p	hsa-miR-608	hsa-miR-449b-5p	hsa-miR-449a	hsa-miR-328-5p
hsa_circRNA_102399	up 2.0267379	hsa-miR-329-5p	hsa-miR-500a-3p	hsa-miR-591	hsa-miR-138-5p	hsa-miR-431-3p
hsa_circRNA_103636	up 2.4697332	hsa-miR-520a-5p	hsa-miR-617	hsa-miR-15b-3p	hsa-miR-890	hsa-miR-103a-2-5p
hsa_circRNA_103637	up 2.4114317	hsa-miR-33a-3p	hsa-miR-520a-5p	hsa-miR-617	hsa-miR-15b-3p	hsa-miR-890
hsa_circRNA_103944	up 2.6690606	hsa-miR-20a-3p	hsa-miR-514a-5p	hsa-miR-33a-5p	hsa-miR-26a-5p	hsa-miR-495-3p
hsa_circRNA_104600	up 2.1080593	hsa-miR-609	hsa-miR-758-5p	hsa-miR-548c-3p	hsa-miR-592	hsa-miR-452-3p
hsa_circRNA_104950	up 2.1982182	hsa-miR-766-3p	hsa-miR-367-5p	hsa-miR-9-5p	hsa-miR-770-5p	hsa-miR-504-5p
hsa_circRNA_400012	up 2.6538905	hsa-let-7e-5p	hsa-let-7c-5p	hsa-let-7d-5p	hsa-let-7b-5p	hsa-let-7f-5p
hsa_circRNA_000094	down 3.962793	hsa-miR-98-5p	hsa-let-7f-5p	hsa-miR-370-5p	hsa-let-7e-5p	hsa-let-7a-5p
hsa_circRNA_000676	down 2.747486	hsa-miR-330-5p	hsa-miR-619-5p	hsa-miR-665	hsa-miR-326	hsa-miR-342-5p
hsa_circRNA_000750	down 2.075174	hsa-miR-513a-3p	hsa-miR-370-3p	hsa-miR-577	hsa-miR-153-5p	hsa-miR-619-5p
hsa_circRNA_000943	down 2.589559	hsa-miR-619-5p	hsa-miR-665	hsa-miR-30b-3p	hsa-let-7e-5p	hsa-let-7c-5p
hsa_circRNA_001401	down 3.231116	hsa-miR-619-5p	hsa-miR-130b-5p	hsa-miR-211-5p	hsa-miR-29b-2-5p	hsa-miR-665
hsa_circRNA_002117	down 2.870608	hsa-miR-185-3p	hsa-miR-371a-3p			
hsa_circRNA_100079	down 4.770937	hsa-miR-627-3p	hsa-miR-22-5p	hsa-miR-651-5p	hsa-miR-152-5p	hsa-miR-146a-3p
hsa_circRNA_100272	down 2.681527	hsa-miR-382-5p	hsa-miR-568	hsa-miR-629-3p	hsa-miR-152-5p	hsa-miR-380-3p
hsa_circRNA_100273	down 2.405354	hsa-miR-380-3p	hsa-miR-141-5p	hsa-miR-329-5p	hsa-miR-21-3p	hsa-miR-30c-5p
hsa_circRNA_100302	down 3.974205	hsa-miR-147a	hsa-miR-485-5p	hsa-miR-432-5p	hsa-miR-597-3p	hsa-miR-758-5p
hsa_circRNA_100319	down 4.378096	hsa-miR-573	hsa-miR-520g-5p	hsa-miR-877-5p	hsa-miR-27a-3p	hsa-miR-621
hsa_circRNA_100433	down 2.825542	hsa-miR-762	hsa-miR-637	hsa-miR-93-3p	hsa-miR-377-5p	hsa-miR-328-5p
hsa_circRNA_100633	down 2.41089	hsa-miR-627-3p	hsa-miR-199b-5p	hsa-miR-450b-3p	hsa-miR-769-3p	hsa-miR-154-5p
hsa_circRNA_100657	down 2.888380	hsa-miR-450b-3p	hsa-miR-619-3p	hsa-miR-486-3p	hsa-miR-769-3p	hsa-miR-619-5p
hsa_circRNA_100790	down 3.416227	hsa-miR-20b-3p	hsa-miR-150-3p	hsa-miR-133a-5p	hsa-miR-509-3p	hsa-miR-485-5p
hsa_circRNA_100844	down 3.40454	hsa-miR-320a	hsa-miR-320b	hsa-miR-497-3p	hsa-miR-22-3p	hsa-miR-98-5p

hsa_circRNA_101055	down 4.522587	hsa-miR-383-3p	hsa-miR-766-3p	hsa-miR-570-3p	hsa-miR-145-3p	hsa-miR-204-3p
hsa_circRNA_101170	down 2.537399	hsa-miR-622	hsa-miR-615-5p	hsa-miR-766-3p	hsa-miR-328-3p	hsa-miR-29c-5p
hsa_circRNA_101175	down 3.033083	hsa-miR-374a-3p	hsa-miR-124-5p	hsa-miR-181b-5p	hsa-miR-181d-5p	hsa-miR-125b-1-3p
hsa_circRNA_101226	down 2.434792	hsa-miR-616-3p	hsa-miR-619-5p	hsa-miR-20b-3p	hsa-miR-562	hsa-miR-493-5p
hsa_circRNA_101258	down 2.036035	hsa-miR-541-5p	hsa-miR-876-5p	hsa-miR-29b-2-5p	hsa-miR-140-5p	hsa-miR-659-5p
hsa_circRNA_101369	down 2.797086	hsa-miR-500a-3p	hsa-miR-422a	hsa-miR-619-3p	hsa-miR-502-3p	hsa-miR-365a-5p
hsa_circRNA_101370	down 2.084251	hsa-miR-196b-3p	hsa-miR-500a-3p	hsa-miR-422a	hsa-miR-619-3p	hsa-miR-134-5p
hsa_circRNA_101531	down 3.797924	hsa-miR-100-3p	hsa-miR-584-5p	hsa-miR-21-5p	hsa-miR-329-3p	hsa-miR-632
hsa_circRNA_101645	down 2.083552	hsa-miR-552-3p	hsa-miR-134-5p	hsa-miR-377-5p	hsa-miR-106b-3p	hsa-miR-662
hsa_circRNA_101850	down 2.037292	hsa-miR-127-5p	hsa-miR-449a	hsa-miR-449b-5p	hsa-miR-34c-5p	hsa-miR-378a-5p
hsa_circRNA_101886	down 2.664644	hsa-miR-578	hsa-miR-367-5p	hsa-miR-188-3p	hsa-let-7a-2-3p	hsa-miR-1301-3p
hsa_circRNA_102119	down 2.731236	hsa-miR-22-5p	hsa-miR-503-3p	hsa-miR-524-3p	hsa-miR-525-3p	hsa-miR-548c-3p
hsa_circRNA_102126	down 3.379792	hsa-miR-580-3p	hsa-miR-605-5p	hsa-miR-647	hsa-miR-376a-2-5p	hsa-miR-449b-3p
hsa_circRNA_102146	down 2.050997	hsa-miR-670-5p	hsa-miR-892a	hsa-miR-450a-1-3p	hsa-miR-125b-5p	hsa-miR-410-5p
hsa_circRNA_102195	down 3.447775	hsa-miR-449c-5p	hsa-miR-328-3p	hsa-miR-193b-3p	hsa-miR-658	hsa-miR-2113
hsa_circRNA_102225	down 2.995241	hsa-miR-520g-3p	hsa-miR-520h	hsa-miR-17-3p	hsa-miR-422a	hsa-miR-764
hsa_circRNA_102367	down 2.056405	hsa-miR-15a-5p	hsa-miR-561-3p	hsa-miR-659-3p	hsa-miR-203a-5p	hsa-miR-217
hsa_circRNA_102492	down 2.475824	hsa-miR-30c-1-3p	hsa-miR-497-5p	hsa-miR-15b-5p	hsa-miR-601	hsa-miR-597-3p
hsa_circRNA_102619	down 2.173889	hsa-miR-452-5p	hsa-miR-874-3p	hsa-miR-218-1-3p	hsa-miR-767-5p	hsa-miR-218-2-3p
hsa_circRNA_102645	down 2.438340	hsa-miR-26a-1-3p	hsa-miR-26a-2-3p	hsa-miR-361-3p	hsa-miR-214-3p	hsa-miR-1323
hsa_circRNA_102765	down 2.135364	hsa-miR-758-5p	hsa-miR-766-3p	hsa-miR-574-5p	hsa-miR-202-5p	hsa-miR-125a-3p
hsa_circRNA_102817	down 2.172231	hsa-miR-331-5p	hsa-miR-298	hsa-miR-296-3p	hsa-miR-30c-1-3p	hsa-miR-588
hsa_circRNA_103198	down 3.415381	hsa-miR-548a-3p	hsa-miR-323a-5p	hsa-miR-888-3p	hsa-miR-514a-3p	hsa-miR-501-3p
hsa_circRNA_103384	down 2.257482	hsa-miR-520f-3p	hsa-miR-106b-5p	hsa-miR-542-3p	hsa-miR-2113	hsa-miR-17-5p
hsa_circRNA_103634	down 4.569931	hsa-miR-95-5p	hsa-miR-100-3p	hsa-miR-340-5p	hsa-miR-140-5p	hsa-miR-150-3p
hsa_circRNA_103655	down 2.067453	hsa-miR-766-5p	hsa-miR-298	hsa-miR-323a-5p	hsa-miR-138-5p	hsa-miR-486-3p
hsa_circRNA_103675	down 3.811865	hsa-miR-362-3p	hsa-miR-96-5p	hsa-miR-103a-3p	hsa-miR-107	hsa-miR-615-3p
hsa_circRNA_103750	down 2.124922	hsa-miR-15a-3p	hsa-miR-338-3p	hsa-miR-382-5p	hsa-miR-218-1-3p	hsa-miR-329-3p
hsa_circRNA_103838	down 3.766406	hsa-miR-519c-3p	hsa-miR-106b-5p	hsa-miR-655-3p	hsa-miR-106a-5p	hsa-miR-17-5p
hsa_circRNA_104227	down 4.085053	hsa-miR-329-3p	hsa-miR-593-5p	hsa-miR-641	hsa-miR-103a-2-5p	hsa-miR-19b-1-5p
hsa_circRNA_104508	down 3.401752	hsa-miR-770-5p	hsa-miR-345-5p	hsa-miR-488-3p	hsa-miR-595	hsa-miR-450b-5p
hsa_circRNA_104543	down 4.926867	hsa-miR-196b-3p	hsa-miR-1298-3p	hsa-miR-345-3p	hsa-miR-1-3p	hsa-miR-1224-3p
hsa_circRNA_104575	down 2.800558	hsa-miR-519e-5p	hsa-miR-519d-5p	hsa-miR-200b-3p	hsa-miR-145-5p	hsa-miR-515-5p

hsa_circRNA_104816	down 2.022495	hsa-miR-561-5p	hsa-miR-140-3p	hsa-miR-122-5p	hsa-miR-505-3p	hsa-miR-612
hsa_circRNA_104904	down 2.960470	hsa-miR-887-5p	hsa-miR-891b	hsa-miR-10b-3p	hsa-miR-409-5p	hsa-miR-575
hsa_circRNA_104941	down 2.460433	hsa-miR-876-3p	hsa-miR-541-5p	hsa-miR-330-3p	hsa-miR-92a-2-5p	hsa-miR-1271-3p
hsa_circRNA_104952	down 2.910698	hsa-miR-660-3p	hsa-miR-877-3p	hsa-miR-200b-3p	hsa-miR-145-3p	hsa-miR-96-5p
hsa_circRNA_400029	down 2.302145	hsa-miR-181b-2-3p	hsa-miR-744-5p	hsa-let-7a-3p	hsa-miR-125a-3p	hsa-miR-597-5p
hsa_circRNA_400068	down 2.760959	hsa-miR-1271-5p	hsa-miR-210-5p	hsa-miR-96-5p	hsa-miR-671-5p	hsa-miR-661
